# Transcatheter aortic valve replacement beneficial in patients with severely reduced left ventricle ejection fraction: does the type of valve also matter?

**DOI:** 10.1002/ehf2.14902

**Published:** 2024-06-05

**Authors:** Marcin Protasiewicz

**Affiliations:** ^1^ Department of Cardiology, Institute of Heart Diseases Wroclaw Medical University Wroclaw Poland

Aortic stenosis is a disease mainly affecting older patients, and most of them have preserved left ventricle ejection fraction.^1^ In this issue of ESC Heart Failure, Matta et al. analyse data from the FRANCE‐TAVI Registry to assess the safety, efficacy, and outcomes of transcatheter aortic valve replacement (TAVR) in patients with severe cardiac dysfunction.^2^ In the study, the group of 157 patients with left ventricular ejection fraction (LVEF) below ≤35% was compared with their 820 counterparts with normal cardiac function.

Data on the outcomes of TAVR in patients with severe LV dysfunction are sparse, even though in daily clinical practice, such patients may represent up to 13% of those undergoing TAVR.^3^ Many factors may predict or influence the long‐term outcomes after the TAVI identified already.^4^, ^5^, ^6^, ^7^ Matta's retrospective, single‐centre cohort study offers valuable insights into a group of patients that have often been poorly represented or excluded in previous trials and analyses.

The study's primary endpoint was to assess the success rate, risk of complications, changes in LVEF, and survival post‐TAVR in patients with severely reduced versus preserved LVEF. The secondary endpoint was to compare survival outcomes post‐TAVR in patients with LVEF ≤35% treated with self‐expanding valves (SEV) versus balloon‐expandable valves BEV.

Characteristics of patients and TAVR procedures in those with LVEF <35% and >50% differed by several variables that may have a prognostic impact, but mainly in favour of the normal LV group. However, during a mean follow‐up of more than 3 years, no differences in all‐cause mortality were observed between both groups at different time points. There is conflicting information on whether LVEF is a risk factor for poor TAVR outcome.^8^, ^9^, ^10^, ^11^ Matta et al. observation indicates that baseline left ventricular function may be not the main dominant predictor of postoperative prognosis, and patients with severely depressed LVEF may benefit from TAVR comparably to patients with preserved LVEF.

An interesting analysis of the outcomes of patients with severe left ventricular dysfunction undergoing TAVR was provided by Witberg et al.^12^ The authors demonstrated that regardless of baseline LVEF, it is LV recovery after TAVR and the extent of this recovery that determined mid‐term mortality in patients with severe AS. Patients with impaired baseline LVEF but complete LV recovery after the procedure had similar mid‐term mortality to those without severe LV dysfunction before TAVR. Predictors of LV recovery after TAVR, including an absence of prior myocardial infarction and high aortic valve gradients (>40 mmHg), also emphasize that LV recovery may not occur in all patients after TAVR, which is indeed observed in previous studies (*Table* *1*).

**Table 1:** Observations on left ventricular recovery after TAVR

Patients' baseline LVEF characteristics	Study	No. of patients	% of patients with LV recovery (EF increase >10%)	Positive predictors of LV recovery HR (95% CI)
<50%	PARTNER Cohort A Elmariah et al.^8^ (2013)	*N* = 108	51.6	Mean AVG (per mmHg): 1.03 (1.01–1.06)
	PARTNER 1, 2 and S3 Kolte et al.^13^ (2022)	*N* = 659	32.8	SVI: 1.03 (1–1.06) BMI: 1.06 (1.02–1.1)
<40%	Medtronic CoreValve U.S. Pivotal Trial Dauerman et al.^14^ (2016)	*N* = 156	62.2	Mean AVG > 40 mmHg: 4.59 (1.76–11.96)
<30%	AMTRAC Registry Witberg et al.^12^ (2023)	*N* = 914	59.5	Mean AVG (per mmHg): 1.02 (1.01–1.04)

AVG, aortic valve gradient; BMI, body mass index; CI, confidence interval; EF, ejection fraction; HR, hazard ratio; LV, left ventricle; MI, myocardial infarction; SVI, stroke volume index; TAVR transcatheter aortic valve replacement.

The results regarding the effect of valve type on prognosis in the LVEF<35% group are intriguing and potentially unexpected. Previous analyses indicated a poorer prognosis, including higher mortality, in patients receiving self‐expanding valves.^15^, ^16^ However, the authors of the present study present data that contradicts this concept. According to their observations, self‐expanding valves confer a significant prognostic benefit in patients with LVEF <35%, contrasting with outcomes in patients with LVEF >50%. These observations require further investigation as they may reveal a previously unreported pattern. Notably, it is interesting that the publications showing these completely contrasting results use data from the same FRANCE‐TAVI registry. However, it should be acknowledged that the current publication is based on data from a single centre, whereas the prior study was not stratified according to LVEF.

Slightly on the fringes of the ongoing discourse regarding the influence of habits and procedural techniques on long‐term outcomes, an article based on AMTRAC Registry data sheds light on an interesting aspect. The authors indicated that a significant interaction could be noted between center valve preference and late mortality.^17^ Periprocedural outcomes and two‐year mortality appear to be compromised when TAVR procedures are conducted using SEVs at centers predominantly favouring BEVs. While I am not implying that a similar phenomenon occurred in this situation, it does indicate the need for cautious interpretation of single‐centre and retrospective data.

Matta et al. observe the early change in LVEF from 29.2 ± 5.5 to 37.4 ± 10.8 post‐TAVR in patients with LVEF ≤35% before hospital discharge. An increase in EF has been shown to be an independent factor affecting survival. Taking into account the results from the AMTRAC Registry and the above observations, it can be assumed that, as perceived by the authors, the lower gradient after TAVR in the SEV group may stem from the larger effective orifice area of the SEV, leading to a greater increase in LVEF (although not statistically significant). This is reasonable, but a comprehensive understanding requires information on the prevalence of previous myocardial infarctions in both SEV and BEV groups. Put differently, it is crucial to ascertain the proportion of patients whose initial heart damage was derived primarily from a valvular defect versus those affected by post‐myocardial fibrosis.

It appears that the most significant observations of the current study are, as indicated by the authors themselves and consistent with existing data, that patients with severely impaired LVEF demonstrate a similar procedural success rate, periprocedural complications risk, and survival outcome to those with normal cardiac function. The results regarding the influence of valve type in patients with LVEF<35% can be considered hypothesis‐generating and worthy of further observation. Thus far, data to guide valve selection in patients with severely depressed LVEF have been lacking.

Despite some limitations (such as a higher proportion of patients treated using a transfemoral approach in the SEV subgroup) and remaining questions, Matta et al. should be commended for their efforts to understand the influence of the TAVR procedure in a high‐risk and understudied population. Perhaps we should also consider the utility of imaging studies in assessing the burden of myocardial fibrosis before TAVR to enhance patient selection and prognosis.

## Conflict of interest

I have no conflict of interests.
